# Assessment of stereoscopic optic disc images using an autostereoscopic screen – experimental study

**DOI:** 10.1186/1471-2415-8-13

**Published:** 2008-07-23

**Authors:** Maged S Habib, James A Lowell, Nick S Holliman, Andrew Hunter, Daniella Vaideanu, Anthony Hildreth, David HW Steel

**Affiliations:** 1 Ophthalmology Department, Sunderland Eye Infirmary, Sunderland, UK; 2 Computer Science Department, Durham University, UK; 3 Department of computing and Informatics, Lincoln University, UK; 4 School of clinical Medical Science, University of Newcastle, UK

## Abstract

**Background:**

Stereoscopic assessment of the optic disc morphology is an important part of the care of patients with glaucoma. The aim of this study was to assess stereoviewing of stereoscopic optic disc images using an example of the new technology of autostereoscopic screens compared to the liquid shutter goggles.

**Methods:**

Independent assessment of glaucomatous disc characteristics and measurement of optic disc and cup parameters whilst using either an autostereoscopic screen or liquid crystal shutter goggles synchronized with a view switching display. The main outcome measures were inter-modality agreements between the two used modalities as evaluated by the weighted kappa test and Bland Altman plots.

**Results:**

Inter-modality agreement for measuring optic disc parameters was good [Average kappa coefficient for vertical Cup/Disc ratio was 0.78 (95% CI 0.62–0.91) and 0.81 (95% CI 0.6–0.92) for observer 1 and 2 respectively]. Agreement between modalities for assessing optic disc characteristics for glaucoma on a five-point scale was very good with a kappa value of 0.97.

**Conclusion:**

This study compared two different methods of stereo viewing. The results of assessment of the different optic disc and cup parameters were comparable using an example of the newly developing autostereoscopic display technologies as compared to the shutter goggles system used. The Inter-modality agreement was high. This new technology carries potential clinical usability benefits in different areas of ophthalmic practice.

## Background

Early detection of progressive glaucomatous optic disc damage is essential in the management of patients with glaucoma. Accurate assessment of the optic disc can reveal early structural changes that precede field changes. In spite of recent developments in imaging techniques of the optic disc – such as the scanning laser tomography and optic coherence tomography-, digital stereoscopy of the optic disc is still considered the gold standard against which other technologies are evaluated [1].

The use of the stereo photographic images of the optic disc is common in glaucoma clinics in the United Kingdom. The images are usually displayed simultaneously on high-resolution computer screens and viewed using a hand held stereo viewer or liquid-crystal shutter goggles. These techniques have their limitations as the viewer has to wear- or be close to – some device to separate the left and right views, together with limited head freedom, and dimness of the displayed stereo image.

Autostereoscopic displays represent a relatively new technology that do not require the observer to wear any device to separate the left and right views and instead present them directly to the correct eye offering potential benefits in ease of use in clinic settings

This study was designed to assess whether the stereoviewing performance of an autostereoscopic screen would provide equivalent clinical and diagnostic accuracy as compared to using liquid crystal shutter goggles working in synchronisation with a view switching display, when viewing stereoscopic optic disc images

## Methods

Sixty optic disc stereo-images were randomly selected from the database of patients who had attended the glaucoma clinic at Sunderland Eye Infirmary- patients with glaucoma, suspect glaucoma and normals were included. The images were 512 × 512 digital monochromatic sequential stereo-photographic optic disc images captured by the DISCAM optic disc camera (Marcher Enterprises Ltd, Hereford) following pharmacological mydriasis as part of the patients' routine care. As the main aim of this study was to compare the quality of stereo viewing using the two modalities, poor quality images due to poor illumination or images with large vertical shift on the screen between the stereo pairs, were excluded. Ethical committee approval was obtained.

The images were displayed in two ways, using a custom designed program written in Borland C++ Builder using the Open GL graphics libraries. The first modality used a shuttering goggle system with a Dell Ultra-scan P1110 21 inch monitor, an Oxygen GVX1 video card and a pair of Stereo-graphics Crystal Eye CE-3 polarized liquid crystal shutter goggles [2]. The Oxygen GVX1 card, in conjunction with Open GL, was able to display sequential stereos at half the maximum refresh rate, by interleaving presentations of the left and right images and synchronously controlling the stereo goggles through a mounted infrared emitter. Given that the P1110 monitor is capable of refresh rates up to 120 Hz, but at 100 Hz with the screen resolution used, we achieved a 50 Hz refresh in stereo mode. The result was satisfactory from the point of view of flicker, and avoided the 50% reduction in vertical resolution suffered by some other shuttering goggle stereo set ups. However, it did still suffer from other problems inherent in the shuttering goggle approach – in particular; variable cross talk between left and right images peaking at 15% [3]; and a 68% reduction in brightness caused by the shuttering effect [2].

Secondly, the stereo-images were displayed on an autostereoscopic screen; a Dimension Technology Inc. 2015 XLS virtual window 15-inch 2D/3D screen [4]. The auto-stereo screen is a flat-panel LCD with a rear illumination optical system that when switched to 3D mode allows a stereoscopic pair to be presented to the viewer at a specific position without requiring the viewer to wear any special glasses. When the observer views the screen at the display's optimum viewing position, "sweet spot", they see half the pixels from the display in the left eye and half the pixels in the right eye [5].

**Figure 1:**
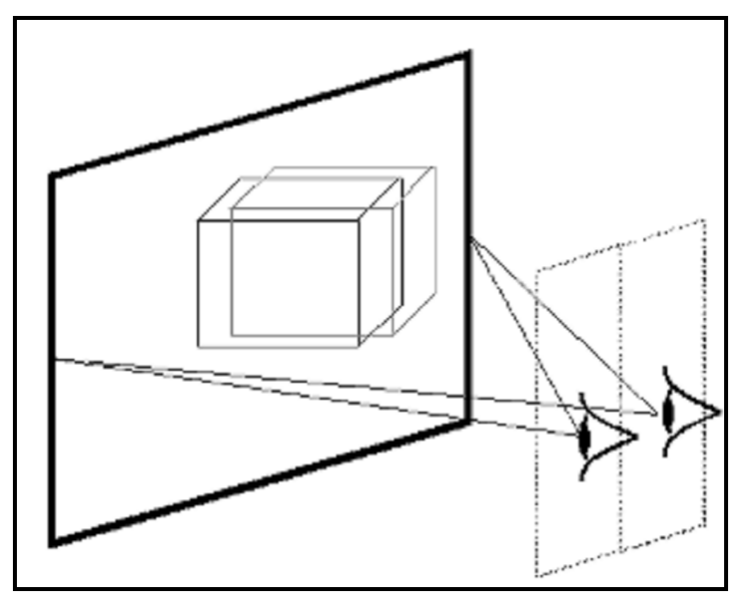
**An example of a two-view autostereoscopic display**. Two-view displays generate the two views for the left and right eyes in two viewing windows in space. These are primarily visible from a central viewing position and the user may have 20 – 30 mm of movement around the central viewing position before they lose the 3D effect.

The 2015 XLS display was driven by placing the stereoscopic image pair side- by side in a single image; this required each image to be shrunk by 50% horizontally. Electronics in the display then interleaved the two images in real time to achieve the required alternate column, interleaving pattern for the 2015 XLS 3D mode. The interleaved images were then visible as completely separate left and right images to the viewer at the display's sweet spot. The display does have an unpublished fixed level of cross talk, although this is at a lower level than the peak of the Crystal-Eyes display. The driver video rate for the 2015 XLS is 60 Hz and although the LC material in the display will not respond as fast as this, for this application using still images, this was not relevant. As a consequence unlike the shutter goggles there was no flicker problem with the 2015 XLS. (Figure 1)

The stereoscopic and auto-stereoscopic displays clearly have differing physical and optical characteristics and our evaluation sought to determine if the differences between the two modalities made qualitative or quantitative differences to performance in assessing various characteristics and different measurements in stereoscopic optic disc images. This was assessed in two ways;

Firstly, quantitative optic disc parameters were analyzed in both modalities using a mouse-controlled cross-shaped cursor and custom designed software. The program allowed the user to first adjust the vertical and horizontal offsets between the two images to achieve a good stereo effect and then a three dimensional cursor was used to mark up the images. The cursor was displayed using the standard "exclusive-OR" approach, where the intensity of grey-scale pixels in the cursor was set to the value 255-g, where g is the pixel value in the covered pixel. Generally speaking this was effective, although we did occasionally observe that, where the left and right image' intensities varied significantly (because of lighting variations), significant differences in the cursor colour could evoke a disassociation, so that two separate crosses were perceived. The cursors depth could be adjusted (changing the disparity between left and right display of the cursor): after adjustment the cursor appeared confined within a chosen plane. This gave a good, consistent stereo effect provided that the cursor was kept in a position where it was correctly perceived to be at the retinal surface.

In each setting the cursor depth was first adjusted to the plane of the outer edge of the optic disc (optic disc rim) whilst stereo viewing. The optic disc edge was then traced with the cursor, clicking to place a contour, which was displayed in the cursor plane. The mouse controlled cursor's depth was then adjusted to the plane at which the disc vessels first deviated posteriorly, and the edge of the optic cup was then traced at that plane, placing a second contour.

After familiarization with these assessment techniques using an additional training set of 10 images, the test images were presented randomly to two observers. The two observers assessed the selected images independently on two separate occasions two months apart (at each time point half the images were viewed using the autostereoscopic system and half using the goggles system to avoid bias) In most cases, the observers needed to adjust the vertical and horizontal offset of the stereo pairs to ensure optimum quality of stereo viewing. The observers were masked to their previous/and other observer's assessments.

Once saved, the computer system then counted the exact pixel number included within each plotted disc and cup areas. The overall cup/disc area ratio was determined as the ratio between the two plotted areas. The vertical cup/disc ratio was determined by calculating the ratio between the height of the disc and cup plotted areas in the vertical meridian. The areas were evaluated for agreement and overlapping between the two viewing modalities.

Secondly, a third independent assessor graded the optic disc appearances on a 5-point scale for the probability of glaucoma as described by Greaney et al [6]. Each optic disc was thus graded as 1(definitely normal), 2(probably normal), 3(undecided), 4(probably glaucoma), and 5(definitely glaucoma). Criteria for allocation to these categories was based on the presence of neuroretinal rim thinning, notching, undermining of optic disc cup edge, nerve fibre layer defects and optic disc haemorrhages [7]. Each disc was also graded in a dichotomous manner as glaucomatous or non-glaucomatous.

The overall optic disc classification grading together with the quantitative disc parameters' scores were then analyzed to assess inter-modality variability.

### Statistical Analysis

Basic statistical analysis was undertaken using SPSS Version 14.0. Observer agreement was assessed via StatXact Version 6.0, whilst S-Plus Version 6.2 professional was used for graphing. Distributions were confirmed as normal via the Shapiro-Wilk test prior to basic parameters (means and standard deviations) being calculated. Agreement was assessed via the weighted kappa test (with 95% confidence intervals) and via Bland Altman plots.

## Results

A total of 60 optic disc stereo-images were included (38 Right and 22 Left). There were 36 males and 24 females. Their age ranged from 45 to 72 years. (Mean = 62 years). Patients were diagnosed with glaucoma (29), ocular hypertension (11), or glaucoma suspect (20).

The inter-modality agreements for observers were assessed using the weighted Kappa coefficient. Landis and Koch [8] offer the following guidelines for interpretation of the weighted kappa coefficient: <0.20 = poor agreement, 0.21 – 0.40 = fair agreement, 0.41 – 0.60 = moderate agreement, 0.61 – 0.80 = good agreement, 0.81 – 1.0 – very good agreement.

The estimates for the overall cup/disc ratio (CDR), and vertical cup/disc ratio (vCDR) were compared. The inter-modality agreement was good for each observer; the average kappa coefficient was 0.78 for observer 1 (p-value <0.0001), and 0.81 for observer 2 (p-value <0.00001). (Table 1)

**Table 1:** Inter-modality agreement for cup-disc ratios

Observer 1 (Autostereoscopic screen versus Shutter Goggles)				Observer 2 (Autostereoscopic Screen versus Shutter Goggles)			
	Weighted Kappa	95% Confidence Interval	Pearson Correlation Coefficient		Weighted Kappa	95% Confidence Interval	Pearson Correlation Coefficient

CDR	0.78	0.65 – 0.91	0.84	CDR	0.83	0.73 – 0.92	0.88
vCDR	0.82	0.73 – 0.91	0.86	vCDR	0.86	0.78 – 0.94	0.88

CDR = overall cup/disc ratio.

vCDR = Vertical cup/disc ratio

**Figure 2:**
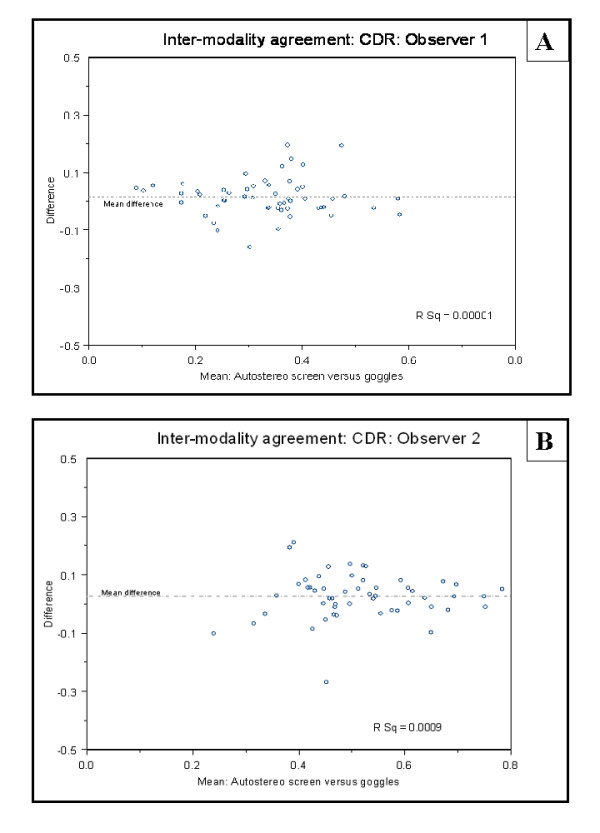
**Bland Altman plots showing the inter-modality agreement of the overall estimated Cup/disc ratio**. A: Inter-modality agreement for observer 1. B: Inter-modality agreement for observer 2.

Using Bland Altman Plots the inter-modality agreement fell within two standard deviations for both observers in more than 95% of cases. Importantly there was no evidence of fixed or proportional bias. (Figure 2)

The plotted cup and disc areas were evaluated for agreement and overlapping between viewing modalities. Observer 1 scored an agreement matching percentage of 97% and 91% for the optic disc and cup areas respectively, while Observer 2 scored an inter-modality agreement of 94% and 89% for the same areas (Table 2)

**Table 2:** Inter-modality agreement for area measurements expressed as Pearson correlation coefficient (p value)

	Observer 1 (Autostereoscopic screen versus Shutter Goggles)	Observer 2 (Autostereoscopic screen versus Shutter Goggles)
Disc Area	0.97 (p < 0.001)	0.71 (p < 0.001)
Cup Area	0.92 (p < 0.001)	0.89 (p < 0.001)

Using Bland Altman Plots, in more than 95% of cases the inter-modality agreement fell within two standards. Again there was no evidence of fixed or proportional bias.

The inter-modality kappa coefficient for the dichotomous grading of glaucoma/non glaucoma was perfect at 1.0. The weighted Kappa coefficient for the five-point grading was very good at 0.97 (95% CI 0.95 to 0.99) with 51 of the 60 cases having exact agreement, i.e. there was exact agreement 85% of the time (95% CI 73% to 93%). None of the nine cases where the five point scale differed had a greater than one point difference. In five patients the grade was higher when using the autostereoscopic screen and in four it was higher using the goggles system suggesting no systematic bias.

## Discussion

Careful and detailed assessment of the optic nerve head is still considered to be the most important sign for diagnosis of glaucomatous progression [9]. In early glaucoma, up to 20–50% of the optic nerve can be lost before any reproducible visual field defect is identified [10] and retinal nerve fiber layer RNFL defects can be only detectable after a 50% loss of neural tissue in a given area [11]. Despite the recent advances of computerized optic disc analysis, fundal biomicroscopic examination or stereo viewing of stereoscopic photographic images remains a gold standard for evaluation and monitoring of the optic disc.

Various studies in the past attempted to compare stereoscopic and non- stereoscopic cup/disc C/D assessments. Results have been equivocal; some studies have shown the stereoscopic C/D ratio measurements to be larger [12-14], equal [15] or smaller than non-stereoscopic images [1]. However recently, Morgan et al reported lower estimates of neuroretinal rim width and higher levels of inter-observer agreement with stereoscopic assessments as compared to monoscopic assessments [16] reinforcing the value of stereoscopic assessment

In this study our aim has been to compare one example of the newly emerging auto-stereoscopic 3D display technologies, DTI 2015 XLS, with an existing stereoscopic display, CrystalEyes CE-3 shutter goggles. Auto-stereoscopic displays offer the potential for "easier" clinical use, without requiring the user to wear goggles. When assessing any new technique which offers improved clinical convenience it is important to ensure that clinical and diagnostic accuracy is not impaired. Hence we designed the study to ascertain whether the detection of glaucomatous optic disc characteristics, and assessment of quantitative optic disc parameters was equivalent between autostereoscopic screen viewing and a commonly used existing method of stereo viewing. This study was an initial step in the assessment of this new technology. We did not attempt to ascertain the accuracy of auto-stereoscopic displays in diagnosing glaucoma and hence did not perform a sensitivity and specificity analysis on this diagnosis, which relies on other investigations including visual fields.

The three observers, who participated in the study, noted a distinct and definite improvement in the subjective perception of the quality of stereopsis in appreciating the optic cup depth together with an increase in the clarity and contrast of the optic disc rim, and the disc vessels in the viewed stereo images when using the auto stereoscopic display as compared to the shutter goggle modality. The reduced brightness, residual flicker and high peak cross-talk levels, as well as the physical 'discomfort' associated with the shutter goggles are all possible reasons. We achieved a 50 Hz refresh in stereo viewing mode for the shutter goggle system. The result was satisfactory from the point of view of flicker, and avoided the 50% reduction in vertical resolution suffered by some other shuttering goggle stereo set-ups but did have a 68% reduction in brightness caused by the shuttering effect. Unlike the shutter goggles there was no flicker problem with the auto stereoscopic screen set up together with relatively less reduction in brightness.

We acknowledge that the study had a potential bias, as we were unable to mask the observers to the stereo viewing modality used – unfortunately any masking would have affected the differences in the observed stereoscopic effect. Previous studies have assumed that different methods of stereo viewing are equivalent and this is the first study that we are aware of in the ophthalmic literature to compare viewing methods.

Analysis of our results demonstrated a good level of inter- modality agreement of cup disc ratio measurements with an average weighted kappa coefficient of 0.8 for the C/D ratios measurements. There was perfect agreement for categorization of the disc to glaucomatous or non-glaucomatous between the two modalities and very good agreement on a five point glaucomatous grading scale. This suggests that viewing stereoscopic optic disc images with the autostereoscopic screen used do not alter the ability the ability of the observer to assess changes in the optic disc as compared to the shutter goggle system used.

A number of other general issues arose during the study concerning the use of any stereoscopic viewing technique for this task, namely cross talk, the marking of feature locations, such as the edge of the optic cup, and image alignment. We believe these deserve further investigation before clinical use of digital stereoscopic imaging can be fully optimized.

In the shutter goggles system used, inter-channel stereoscopic cross-talk peaks at 15%. The auto stereoscopic display used does have an unpublished fixed level of cross talk and although this is at a level lower than the peak of the Crystal- Eyes display both display modalities have significant cross-talk. This could easily lead to errors in judgment of region boundaries, particularly where the edge is initially of low contrast. It may be that recently available zero cross talk stereoscopic or auto-stereoscopic systems will prove more suited to clinical tasks. These should certainly be used in any comparisons with Gold standard results.

Other studies have reported differences in marking between monoscopic and stereoscopic modes. We believe this may be partly due to imaging issues such as cross talk. For example this could wash out an edge and result in consistent under-estimation of feature size. Alternatively it could extend an edge and result in consistent over-estimation of feature size. This may explain conflicting results in previous studies. Again studies are needed using a modern zero cross- talk display system to investigate this and several other more detailed aspects that arise from mismatches between the geometry of the picking cursor and the camera used to capture the original images

It became clear during the study that the alignment of images from the camera was often poor vertically and horizontally, to the extent that images could be uncomfortable to fuse without manual adjustment. This will significantly affect task performance, particularly in comparative imaging of one patient over time. Auto image alignment is now becoming feasible using software but it may be that this issue is best resolved by manufacturers improving camera design.

## Conclusion

Auto-stereoscopic display technology is rapidly evolving and improving and we anticipate this will lead to additional quantitative benefits using the latest high contrast, low cross-talk, displays. Our results show that viewing of stereoscopic optic disc images using an autostereoscopic screen provides comparable diagnostic and clinical assessment to liquid crystal shutter goggles. Furthermore we believe that autostereoscopic displays have significant clinical usability benefits over goggle based stereoscopic displays.

## Competing interests

The authors declare that they have no financial or any other non-financial competing interests in any of the products mentioned in this manuscript.

The authors confirm that this work received no public or organizational funding

## Authors' contributions

MSH and DV and DHWS carried out the independent viewing processes with the 2 modalities. MSH participated in the study design and drafted the manuscript. NSH, AH and JAL provided the stereoviewing technology and designed the needed computer software. AH performed the statistical analysis. DHWS, the lead investigator, conceived the study, participated in its design and coordination and helped to draft and revise the manuscript. All authors read and approved the final manuscript

## Pre-publication history

The pre-publication history for this paper can be accessed here:


